# Clinical and therapeutic implications of HLAB51-negative status in Pediatric Behçet’s Disease

**DOI:** 10.1016/j.clinsp.2026.101072

**Published:** 2026-07-29

**Authors:** Matheus Santos França, Thales Queiroz Souza, Ricardo Nóbrega Machado, Vinicius Campos Matias, Verena Andrade Balbi, Nadia Emi Aikawa, Katia Tomie Kozu, Clovis Artur Silva, Lucia Maria de Arruda Campos, Adriana Maluf Elias

**Affiliations:** a Instituto da Criança e do Adolescente, Hospital das Clínicas da Faculdade de Medicina da Universidade de São Paulo, São Paulo, SP, Brazil; b Faculdade de Medicina, Universidade de Brasília, Brasília, DF, Brazil; c Faculdade de Ciências Médicas da Santa Casa de São Paulo, São Paulo, SP, Brazil; d Division of Rheumatology, Hospital das Clínicas da Faculdade de Medicina da Universidade de São Paulo, São Paulo, SP, Brazil

*Dear Editor,*

Behçet’s Disease (BD) was first described in 1937 by Hulusi Behçet, although only in the 1970s, Ohno & Dausset reported its association with the presence of HLA-B5.^1^^,^^2^^,^^3^ Several studies have confirmed the strong association between BD and HLA-B51, particularly in patients from the Middle and Far East and those with more severe disease.^4^^,^^5^ Currently, studies have demonstrated that the HLA-B exonic sequence encoding HLA-B51 is the main pathogenic factor in BD.^6^

The prevalence of BD shows a clear geographical gradient, with higher rates along the “Silk Road” region and much lower frequencies in Western Europe and the Americas. The disease usually manifests in the third decade of life, but both early and late onsets are reported. Pediatric BD (pBD) accounts for 2%–21% of cases depending on ethnicity and region.^7^ Diagnosis in children and adolescent is particularly challenging, as symptoms may be incomplete during childhood and evolve over time. To address this, the Pediatric BD group developed classification criteria,^7^ though expert clinical judgment remains essential.

HLA-B51 positivity varies widely among studies comprising different populations. In France, HLA-B5 was present in 7 of 15 patients, although one had HLA-B12.^7^ Reports from India described HLA-B37/B7,^8^ and from Taiwan, it was described the presence of HLA-B56 in a 6-year-old girl with incomplete intestinal BD.^9^ These data suggest that, although HLA-B51 is the strongest risk allele, other HLA alleles may also play a role in disease manifestation.

In Latin America, particularly in Brazil, HLA-B51 was detected in 15.5% of healthy individuals^10^ and data on pBD remain scarce. Given the multiethnic and genetic diversity of the Brazilian population and the scarcity of pediatric data in Latin America, we investigated HLA associations in children and adolescents with BD followed at a tertiary center.

We analyzed 32 cBD patients (diagnosis ≤ 18 years-old) diagnosed according to the consensus classification criteria for pediatric Behçet’s disease^7^ followed at a tertiary pediatric rheumatology center in Brazil. Median age at diagnosis was 4.5 years (0.5–12), 50% were female, 78% self-declared white ethnicity; 5 (16%), brown ethnicity and 2 (6%), black ethnicity. HLA-B51 was identified in 16 patients (50%). Other alleles were identified in 18 patients and included HLA-B15 (n = 8), HLA-B35 (n = 6), HLA-B42 (n = 3), HLA-B53 (n = 3), HLA-B39 (n = 2), HLA-B58 (n = 1), HLA-B57 (n = 1), HLA-B52 (n = 1), and HLA-B14 (n = 1) (Table 1). Two groups were defined according to HLA-B51 positivity. In the positive group: HLA-B44 (n = 3), HLA-B35 (n = 2), HLA-B15 (n = 2), HLA-B52 (n = 1), HLA-B27 (n = 1) e HLA-B53 (n = 1) were observed; whereas in the negative group: HLA-B15 (n = 6), HLA-B 35 (n = 4), HLA-B 42 (n = 3), HLA-B 39 (n = 2), HLA-B 14 (n = 1), HLA-B 53 (n = 2), HLA-B 37 (n = 1), HLA-B 50 (n = 1), HLA-B 58 (n = 1), HLA-B 44 (n = 1), HLA-B 57 (n = 1). Locus B alleles of HLA-B51 positive patients with other allele associate are: B*51: CAHHT (B*41: APTAF), B*51:DWEHV (B*44:DWEHU and B*44:BEMCT), B*51:DWFAC (B*15:DVZUY), B*51:DFTCZ (B*13:CVECJ) and B*51:BHXDT (B*27:BFSEU). Locus B alleles of HLA-B51 negative patients with other allele associate are: B*15:AJFKM (B*35:AJFNW), B*15:BHTPV (B*35:BHTPW), B*15:ZKMW (B*35:AKFJG), B*15:CCSPB (B*15:DNVSD), B*15CVGD2 (B*15 CCWFW), B*15:ATEXY (B*39:ATEXZ), B*39:BEURK (B*42:ASYSV), B*14:DWKPS (B*53:DWKPU), B*42:AMMP (B*37:AGJVP), B*50:EBHVD (B*58:EBHVE), B*44:DFMYZ (B*53:DFMZA), B*35: DFPMW (B*42: CVDGJ), B*35: WBYS (B*57: XSFF), B*57:BYMGD (B*15:BYMGE).

**Table 1:** Demographic, clinical manifestations and treatment in 32 patients with pediatric Behçet disease.

Variables at diagnosis	HLA B51 positive (n = 16)	HLA B51 negative (n = 16)	p-value
	n (%)	n (%)	
**Demographic data**			
Age at diagnosis, years	3 (0.5‒11)	7.5 (0.5‒12)	**0.02**
Currently age, years	15 (8.0‒22)	17 (5.0‒22)	0.72
Disease progression time	7.0 (1.5‒15.3)	6.5 (3.0‒11.5)	0.34
Ethnicity (Caucasian)	14 (88)	11 (69)	0.20
Female sex	11 (69)	5 (31)	**0.04**
**Clinical manifestation**			
Fever	8 (50)	8 (50)	0.57
Oral ulceration	16 (100)	15 (94)	0.50
Genital ulceration	5 (31)	6 (38)	0.50
Arthritis/arthralgia	10 (63)	7 (44)	0.24
**Cutaneous involvement**	3 (19)	5 (31)	0.34
Pseudofolliculitis	1 (6)	5 (31)	0.09
Erythema nodosum	2 (13)	0	‒
Skin ulceration	0	1(6)	‒
**Ocular involvement**	2 (13)	5 (31)	0.20
Uveitis	2 (13)	5 (31)	**0.20**
Vasculitis	2 (13)	4 (25)	0.33
Sequelae injury	0	0	
**Neurological involvement**	3 (19)	5 (31)	0.34
Parenchymal involvement	3 (19)	4 (25)	0.50
Aseptic meningitis	0	3 (19)	‒
**Gastrointestinal involvement**	3 (19)	5 (31)	0.34
**Familial history of autoimmune diseases**	6 (38)	6 (38)	0.59
**Other HLA positive (non-B51)**	6 (38)	12 (75)	**0.04**
**Criteria confirmation**			
PEDBD	3 (19)	7 (44)	0.13
ICBD	7 (44)	9 (56)	0.36
At least, one criteria	4 (25)	2 (13)	0.33
**Treatment**			
Colchicine	14 (88)	15 (94)	0.50
Monotherapy	9 (69)	7 (47)	0.21
Association with other immunosuppressants drugs	3 (19)	9 (56)	**0.05**
Thalidomide	1 (6)	1 (6)	0.76
Glucocorticoid	3 (19)	9 (56)	**0.03**
Azathioprine	3 (19)	11 (69)	**0.006**
Mycophenolate	2 (13)	1 (6)	0.50
Methotrexate	4 (25)	4 (25)	0.69
Cyclosporine	0	1 (6)	‒
Cyclophosphamide	0	0	‒
Immunobiological therapy	0	0	‒
**Death**	0	0	‒

Results are expressed in median (range) and n (%). Continuous data were compared using Mann-Whitney test, and categorical variables with the chi-square or Fisher's exact tests, as appropriate, always as two-sided analyses.

HLA-B51 negative patients were older at diagnosis [7.5 vs. 3 years-old; p = 0.02] and less frequently of female sex (31%vs. 69%; p = 0.04). The presence of other HLA alleles was significantly higher in this group (75%vs. 38%; p = 0.04). Clinical manifestations (oral/genital ulcers, fever, articular, cutaneous, ocular, neurologic and gastrointestinal involvement) and colchicine use were similar between groups (p > 0.05). However, HLA-B51 negative patients more frequently required additional corticosteroids (56%vs. 19%; p = 0.03) and azathioprine (69%vs. 19%; p < 0.05) (Table 1).

In exploratory logistic regression adjusted for age at symptom onset, HLA-B51 positivity remained associated with lower azathioprine use (OR = 0.15, 95% CI 0.02–0.92), whereas age at symptom onset was not associated with treatment requirement. These findings suggest that the greater use of azathioprine among HLA-B51-negative patients was not fully explained by differences in age at disease onset alone. Nevertheless, given the small sample size and retrospective design, these findings should be interpreted as hypothesis-generating rather than evidence of a definitive causal association.

This study has some limitations that should be acknowledged. First, the absence of a healthy control group precludes definitive conclusions regarding disease susceptibility and the independent association of non-HLA-B51 alleles with pBD. Second, the relatively small sample size and exploratory nature of the analyses require cautious interpretation of the findings. Finally, treatment-related analyses may have been influenced by confounding by indication, since therapeutic decisions were based on clinical severity and physician judgment in routine practice.

Our findings indicate that only half of Brazilian pBD carry HLA-B51, while alternative non-B5 HLA alleles were common and associated with older age at diagnosis. These results highlight the genetic heterogeneity of pBD outside the Silk Road and emphasize that the absence of HLA-B51 may be responsible for the delay in disease recognition. Further multicenter studies in Latin America, including healthy control group and whole exome-sequencing, are essential to characterize the genetic landscape of this diverse and multiethnic population.

## Ethical approval

Approved by the institutional ethics committee.

## Funding

FAPESP (Grants 2022/12,925-8 to CAS and NEA and 2023/12,748-1 to LMAC) and 10.13039/501100003593CNPq (Grants 318356/2025-2 to CAS).

## Data availability

Not applicable.

## Declaration of competing interest

The authors declare no conflicts of interest.

## References

[1] Behçet H. (1937). Über rezidivierende, aphthöse, durch ein virus verursachte Geschwüre am Mund, am Auge und an den Genitalien. Dermatol Wochenschr.

[2] Ohno S., Aoki K., Letter Sugiura S. (1973). HLA-B5 and Behçet’s disease. Lancet.

[3] Dausset J. (1974). Genetic basis of Behçet’s disease. Tissue Antigens.

[4] Yazici H., Seyahi E., Hatemi G., Yazici Y. (2018). Behçet syndrome: a contemporary view. Nat Rev Rheumatol.

[5] Maldini C., Lavalley M.P., Cheminant M., de Menthon M., Mahr A. (2012). Relationships of HLA–B51 or B5 genotype with Behçet’s disease clinical characteristics: systematic review and meta-analyses of observational studies. Rheumatology.

[6] Ombrello M.J., Kirino Y., de Bakker P.I.W., Gül A., Kastner D.L., Remmers E.F. (2014). Behçet disease-associated MHC class I residues implicate antigen binding and regulation of cell-mediated cytotoxicity. Proc Natl Acad Sci U S A.

[7] Kone-Paut I., Shahram F., Darce-Bello M., Cantarini L., Cimaz R., Gattorno M. (2016). Consensus classification criteria for pediatric Behçet’s disease. from a prospective observational cohort. PEDBD. Ann Rheum Dis..

[8] Panicker J.N., Vinayan K.P., Ahsan Moosa N.V., Elango E.M., Anand Kumar A. (2007). Juvenile Behçet's disease: highlighting neuropsychiatric manifestations and putative genetic mechanisms. Clin Neurol Neurosurg.

[9] Ke Y.Y., Hwang K.P., Lee I.H., Chai CY. (2003). Behcet's disease in childhood: report of one case. Acta Paediatr Taiwan.

[10] Belem J.M.F.M., Fraga A.M., Andrade L.E.C., Souza A.W.S. (2020). HLA-B*51 and its main subtypes in Brazilian patients with Behçet’s disease. Clin Exp Rheumatol.

